# Radiological Outcome Measures Indicate Advantages of Precontoured Locking Compression Plates in Elderly Patients With Split-Depression Fractures to the Lateral Tibial Plateau (AO41B3)

**DOI:** 10.1177/21514593211043967

**Published:** 2021-10-13

**Authors:** Wolf C. Prall, Thomas Kusmenkov, Maximilian Rieger, Florian Haasters, Hermann O. Mayr, Wolfgang Böcker, Julian Fürmetz

**Affiliations:** 1 Division of Knee, Hip and Shoulder Surgery, Schoen Clinic Munich Harlaching, Academic Teaching Hospital of the Paracelsus Medical University (PMU), Salzburg, Austria; 2 Department of General, Trauma and Reconstructive Surgery, 27192 Munich University Hospital, Ludwig-Maximilians-University (LMU), Munich, Germany; 3 Department of Orthopaedics and Trauma Surgery, 14879 Freiburg University Hospital, Albert-Ludwigs-University, Freiburg, Germany

**Keywords:** adult reconstructive surgery, fragility fractures, geriatric trauma, osteoporosis, osteosynthesis, radiological outcome, Schatzker II, split-depression, tibial plateau fracture, trauma surgery

## Abstract

**Background:**

Split-depression fractures to the lateral tibial plateau (AO41B3) often feature severe joint surface destructions. Precontoured locking compression plates (LCPs) are designed for optimum support of the reduced joint surface and have especially been emphasized in reduced bone quality. A lack of evidence still inhibits their broad utilization in elderly patients. Thus, aim of the present study was to investigate the implant-specific radiological outcomes of AO41B3-fractures in young versus elderly patients.

**Methods:**

The hospital’s database was screened for isolated AO41B3-factures, open reduction and internal fixation (ORIF), and radiological follow-up ≥12 months. CT-scans, radiographs, and patients’ records were analyzed. Patients were attributed as young (18–49) or elderly (≥50 years). Additional subgrouping was carried out into precontoured LCP and conventional implants. The Rasmussen Radiological Score (RRS) after 12 months was set as primary outcome parameter. The RRS postoperatively and the medial proximal tibial angle (MPTA) postoperatively and after 12 months were secondary outcome parameters.

**Results:**

Fifty nine consecutive patients were included (26 young, 38.2 ± 7.8 years; 33 elderly, 61.3 ± 9.4 years). There were no significant differences regarding mean size and depression depth of the lateral joint surface fragments. Prior to implant-specific subgrouping, the radiological outcome measures revealed no significant differences between young (RRS = 7.7 ± 1.7; MPTA = 90.3 ± 2.3°) and elderly (RRS = 7.2 ± 1.7; MPTA = 90.5 ± 3.3°). After implant-specific subgrouping, the radiological outcome revealed significantly impaired results in young patients with conventional implants (RRS_(C)_ = 6.9 ± 1.6, RRS_(LCP)_ = 8.5 ± 1.5, *P* = .015; MPTA_(C)_ = 91.5 ± 1.9°, MPTA_(LCP)_ = 89.1 ± 2.1°, *P* = .01). The effect was even more pronounced in elderly patients, with highly significant deterioration of the radiological outcome measures for conventional implants compared to precontoured LCP (RRS_(C)_ = 5.7 ± 1.6, RRS_(LCP)_ = 8.2 ± .8, *P* < .001; MPTA_(C)_ = 92.6 ± 4.2°, MPTA_(LCP)_ = 89.2 ± 1.4°, *P* = .002).

**Conclusion:**

Utilizing precontoured LCP in the treatment of AO41B3-fractures is associated with improved radiological outcomes. This effect is significant in young but even more pronounced in elderly patients. Consequently, precontoured LCP should closely be considered in any AO41B3-fracture, but especially in elderly patients.

## Introduction

Fractures to the tibial plateau account for approximately 1–2% of all fractures in the general population but for up to 8% of all fractures in the elderly.^1,2^ While younger patients predominately suffer traffic accidents, high impact sport injuries, or falls from greater height, elderly patients often mainly suffer simple falls or low velocity traumata resulting in a tibial plateau fracture. The loss of bone mass and the deterioration of the bone microarchitecture with age contribute to this change of causal injury mechanism. The meta-to epiphyseal zone of the proximal tibia represents a predilection site of osteopenia and osteoporosis with loss of bone mineral density comparable to the lumbar vertebrae.^3^ The manifestation features a reduced trabecular connectivity in the anterior and posterior aspects of the weight bearing areas of the tibial plateaux and a reduced subchondral cortical bone thickness.^4^ Tibial plateau fractures in the elderly are more likely to feature an involvement of the intercondylar eminence.^5^ Furthermore, the comorbidity burden and the presence of preexisting degenerative lesions may contribute to higher rates of symptomatic posttraumatic osteoarthritis in elderly compared to young patients.^6,7^ Higher rates of subsequent total knee arthroplasty secondary to reconstructive surgery also are associated with increasing patient age.^8^ The incidences of total knee replacement for women and patients above 50 years show a substantial increase during the first 5 years after tibial plateau fracture.^9^ And with improved life expectancy, incidences of tibial plateau fractures in elderly patients are probably rising.^10,11^ More than half of all tibial plateau fractures in the elderly are split-depression fractures to the lateral condyle.^12^ These injuries are classified as type II according to Schatzker^13^ or as type 41B3 according to the AO/OTA (www.aofoundation.org).

Surgical treatment is frequently required to restore the articular surface, to provide stability and to re-establish the limb’s mechanical alignment.^14^ Open reduction and internal fixation (ORIF) thereby often combined with void filling according to the bony defect emerging after elevation of the depressed joint surface fragments.^15,16^ The internal fixation can be carried out utilizing conventional implants or precontoured locking compression plates (LCPs). The general recommendation by the AO/OTA still is the utilization of conventional implants such as dynamic compression plates and lag screws. According to the AO/OTA, the utilization of precontoured LCP should be reserved to cases of extreme osteoporosis. Even though precontoured LCP had especially been developed for osteoporosis-associated fractures with poor bone quality, as the design allows for optimum support of the reduced joint surface fragments and angular stable locking of the screws, these implants may also be beneficial in elderly patients in general or even in young patients. But a lack of evidence and conflicting data in the literature hamper the broad utilization of precontoured LCP in split-depression fractures to the lateral tibial plateau in all age groups.

To date, no study has systematically investigated the age-dependent effect of locking versus nonlocking plating on radiological outcome measures of AO41B3-fractures. Thus, the aim of the present study was to determine the Rasmussen Radiological Score (RRS) approximately 12 months after ORIF of split-depression fractures to the lateral tibial plateau separately in young and elderly patients. The RRS postoperatively and the medial proximal tibial angle (MPTA) postoperatively and after 12 months were set as secondary outcome parameters. It was hypothesized that the utilization of precontoured LCP is associated with superior radiological outcome measures in young as well as in elderly patients.

## Materials and Methods

### Patient Groups

All patients ≥18 years of age with tibial plateau fractures and ORIF between January 2010 and December 2016 were retrospectively identified in the hospital’s data base. By means of preoperative X-rays and CT-scans, the fractures were classified according to the AO/OTA classification system (www.aofoundation.org). The following inclusion and exclusion criteria were applied.

Inclusion criteria: AO41B3-fractures to the lateral tibial plateau as classified according to the AO/OTA; ORIF utilizing either precontoured LCP or conventional dynamic compression plates and lag screws; complete radiological assessment encompassing preoperative X-rays and CT scans as well as X-rays taken immediately postoperatively (1. X-ray), approximately 6–8 weeks postoperatively (2. X-ray) and approximately 12 months postoperatively (3. X-ray). Exclusion criteria: Tibial plateau fractures classified other than AO41B3; polytraumatized patients; patients featuring open fractures, pathologic fractures or chain injuries of the affected limb; incomplete radiological assessment.

Age, gender, date of surgery, void filling, and dates of subsequently conducted postoperative radiological examinations were collected from the patients’ records. Initial grouping was conducted regarding the patients’ age. Patients between 18 and 49 years of age were attributed as young and patients ≥50 as elderly. In order to analyze the implant-specific effect on the radiological outcome, further investigations were carried out after subgrouping both age groups according to the implant utilized (precontoured LCP vs conventional implant).

The study was conducted according to the Declaration of Helsinki. The study had been approved by the IRB of the authors’ affiliated institutions (detailed information will be provided after acceptance) and informed written consent was obtained from each patient.

### Fracture Characteristics

The diagnosis of an AO41B3-fracture was confirmed by 3 senior investigators (WCP, TK, and JF) based conventional X-rays in 2 planes and CT-scans prior to ORIF in all patients included. The analyses of the precise fracture characteristics encompassed determining the extended joint surface destruction. To this end, the following parameters were quantified as published previously.^17^ The dimensions of the depressed joint surface fragments were measured in coronal (a) and sagital (b) CT-scan planes; the area (A) of the mainly oval-shaped joint surface fragments was calculated as A=(a/2)×(b/2)×π . The maximum depression depth (c) of the joint surface fragment was measured in sagital CT-scan planes. The approximate bony void volume (V) was calculated as V=A×(2c/3) for the purpose of this study.

### Radiological Outcome Measures

**Figure 1.**
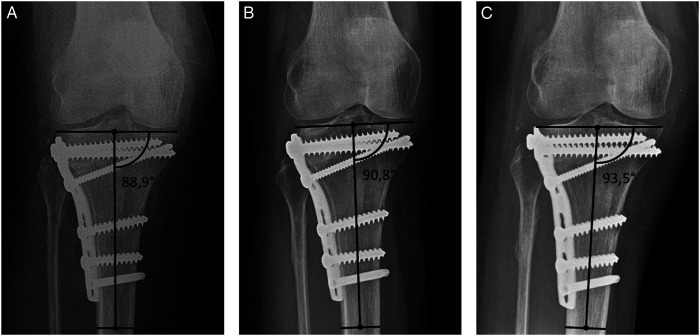
The Rasmussen Radiological Score and the MPTA were evaluated immediately postoperatively (a) as well as approximately six weeks (b) and one year (c) after open reduction and internal fixation.

The X-rays conducted immediately postoperatively and over the course of time were evaluated according to the modified Rasmussen Radiological Score (RRS; Table 1).^18,19^ The varus/valgus angulation was determined as a modified medial proximal tibial angle (MPTA) based on coronal X-ray planes, extrapolating the anatomical tibial axis to the maximum extent possible (Figure 1).^17^ The degree of osteoarthrosis at follow-up was assessed applying the Resnick and Niwoyama criteria (Table 2).^18^ First, the RRS mean numeric values were analyzed. Second, the distribution of the nominal RRS results was evaluated at final follow-up. Accordingly, 9–10 points were considered an excellent, 7–8 points a good, 5–6 points a fair, and <5 points a poor result. Patients that required a total knee arthroplasty (TKA) within the 12 months were defined as treatment failure and were assigned 3 points on the modified RRS at final follow-up.

**Table 1.** Modified Rasmussen Criteria for Radiological Assessment.^18,19^

	Points
Articular depression	
None	3
<5 mm	2
6–10 mm	1
>10 mm	0
Condyle widening	
None	3
<5 mm	2
6–10 mm	1
>10 mm	0
Varus/valgus angulation	
None	3
<10°	2
10–20°	1
>20°	0
Osteoarthrosis	
None/no progression	1
Progression by 1 grade	0
Progression by >1 grade	−1
Excellent	9–10
Good	7–8
Fair	5–6
Poor	<5

**Table 2.** Grading of Osteoarthrosis According to Resnick and Niwoyama.^18^

Grade	
0	None
1	Minimal joint space narrowing Mild sclerosis
2	Moderate narrowing, osteophytes Moderate subchondral sclerosis Moderate bony aberration Intra-articular osseous bodies
3	Marked joint space narrowing Bony collapse Severe subchondral sclerosis Marked deformity Severe bony aberration

### Statistical Analysis

Metric data is presented as mean values and standard deviation. The minimum level of significance was set at *P* ≤ .05. The mean RRS value approximately 12 months postoperatively was set as primary outcome parameter.^18,19^ Previous examinations showed mean RRS values of 8.2 ± 1.2 for precontoured LCP and 6.3 ± 1.7 for conventional implants after 66.1 and 62.3 weeks in age-pooled cohorts.^17^ Based on a probability of less than 5% for type I error and a power of 80%, the sample size was calculated to be 13 per group. Intra- and interrater reliability of the Rasmussen Radiological Score was analyzed in a subset of 15 consecutive cases based on 2 different runs with an interval of 3 months and 2 different raters (WCP and JF). The intraclass correlation coefficients (ICCs) were calculated based on two-way-mixed effects. Further statistical analyses were performed using parametric and nonparametric tests. The Mann–Whitney U Test and the Student’s t-test were used for interval and ratio scaled data. For nominal or ordinal categories, the *χ*^2^ test or the Fisher’s exact test in case of singular low frequencies were applied. Statistical analysis was conducted with the SPSS software package version 25.0 (SPSS, Chicago, Illinois, USA).

## Results

### Patients’ Demographics

**Figure 2.**
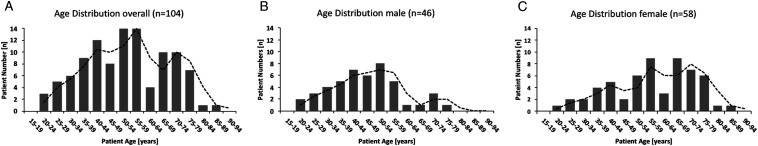
The overall age distribution was evaluated in 104 patients with AO41B3-fractures and ORIF treated at the group’s hospital 2010–2016 (a). The gender-specific age distribution shows that younger patients are predominately male (b) and elderly patients predominately female (c).

Between 2010 and 2016, a total of 104 patients suffered an AO41B3-fracture and underwent ORIF at the group’s hospital. Evaluating patients’ demographics and age distribution revealed that younger patients were predominately male and elderly patients predominately female (Figure 2). After applying the exclusion criteria, a total of 59 patients were finally included (Table 3). The patients’ records verified that postoperative protocols did not differ between the groups. The standardized postoperative protocol encompassed no weight bearing for 6–8 weeks followed by a routinely conducted X-ray examination prior to gradually increasing the load by 20 kg every 2 weeks. There was no routinely conducted X-ray examination with achieving full weight bearing. Range of motion exercises were routinely recommended immediately postoperatively.

**Table 3.** Patients’ Demographics, Fracture Characteristics, and Mean Timepoints of Radiological Follow-up for Overall Comparison of Young and Elderly Prior to Implant-Specific Subgrouping.

	Young (*n* = 26)	Elderly (*n* = 33)	*P*
Age (years)	38.2 (±7.8)	61.3 (±9.4)	n/a
Gender distribution (m: f)	18:8	15:18	.07
Depressed joint area (cm^2^)	4.1 (±.8)	4.7 (±2.0)	.58
Max. depression depth (mm)	10.4 (±6.3)	11.0 (±7.0)	.92
Void volume (mm^3^)	29.6 (±20.0)	36.8 (±29.4)	.69
Void filing (yes: no)	10:16	24:9	*.02*
X-ray (days)	2.4 (±2.3)	2.2 (±1.7)	.79
X-ray (weeks)	6.9 (±2.3)	7.7 (±4.0)	.42
X-ray (weeks)	65.4 (±38.0)	66.0 (±33.5)	.95

### Intra- and Interrater Reliability of the RRS

The intraclass correlation coefficient (ICC) revealed an intrarater reliability of .894 with a 95% confidence interval of .802 to .943. The interrater reliability was .852 with a 95% confidence interval of .723 to .921.

### Young Versus Elderly Prior to Implant-specific Subgrouping

The young group encompassed 26 patients with a mean age of 38.2 ± 7.8 years. The elderly group featured 33 patients with a mean age of 61.3 ± 9.4 years. Across the 2 age groups, there were no significant differences regarding the mean size (4.1 ± 0.8 cm^2^ vs 4.7 ± 2.0 cm^2^; *P* = .58) and depression depth (10.4 ± 6.3 mm vs 11.0 ± 7.0 mm; *P* = .92) of the lateral joint surface fragments or the calculated void volume (29.6 ± 20.0 mm^3^ vs 36.8 ± 29.4 mm^3^; *P* = .69). A void filling was significantly more frequently carried out in the elderly group (*P* = .02). The radiological follow-up took place after 2.4 ± 2.3 days, 6.9 ± 2.3 and 65.4 ± 38.0 weeks in the young and after 2.2 ± 1.7 days, 7.7 ± 4.0 and 66.0 ± 33.5 weeks in the elderly group (Table 3).

**Figure 3.**
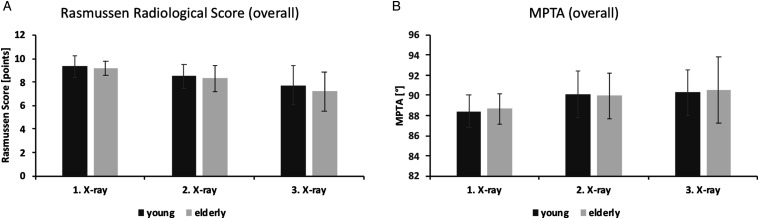
The radiological follow-up of all patients included revealed no significant differences in the Rasmussen Radiological Score (a) or the MPTA (b) between young and elderly prior to implant-specific subgrouping (overall).

The mean RRS value was 9.3 ± .9, 8.5 ± 1.0, and 7.7 ± 1.7 on the first, second, and third X-ray in the young compared to 9.2 ± .6, 8.3 ± 1.1, and 7.2 ± 1.7 in the elderly group. At all 3 timepoints, there were no significant differences between the two age groups. The according MPTAs were 88.4 ± 1.6, 90.1 ± 2.3, and 90.3 ± 2.3° in the young and 88.7 ± 1.5, 90.0 ± 2.3, and 90.5 ± 3.3° in the elderly group. Again, there were no significant differences across the groups (Figure 3). Based on the third X-ray, the distribution of the nominal RRS results (excellent, good, fair, and poor outcome) did not differ significantly between the 2 groups (young: 10, 11, 4, and 1 vs elderly: 5, 19, 6, and 3; *P* = .21).

### Precontoured LCP Versus Conventional Implants in Young Patients

Implant-specific subgrouping of the young patients revealed ORIFs utilizing precontoured LCP and conventional implants in 13 cases of each group. Across the 2 subgroups, no significant differences were found regarding age, gender distribution, the mean size and depression depth of the lateral joint surface fragments, calculated void volume, or frequency of void filling (Table 4).

**Table 4.** Patients’ Demographics and Fracture Characteristics After Implant-Specific Subgrouping of Young and Elderly Patients.

	Young		*p*	Elderly		
	Precont. LCP (*n* = 13)	Conventional (*n* = 13)		Precont. LCP (*n* = 20)	Conventional (*n* = 13)	*P*
Age (years)	38.7 (±7.6)	37.8 (±8.3)	.78	60.2 (±9.8)	62.9 (±8.7)	.42
Gender distribution (m: f)	10:3	8:5	.67	9:11	6:7	.77
Depressed joint area (cm^2^)	4.0 (±.8)	4.3 (±.8)	.39	4.6 (±1.8)	4.9 (±2.2)	.68
Max. depression depth (mm)	12.2 (±7.5)	8.7 (±4.3)	.15	10.6 (±6.7)	11.6 (±7.8)	.7
Void volume (mm^3^)	33.9 (±22.3)	25.3 (±16.4)	.28	35.9 (±30.5)	38.2 (±28.8)	.83
Void filing (yes: no)	6:7	4:9	.42	15:5	9:4	.72

**Figure 4.**
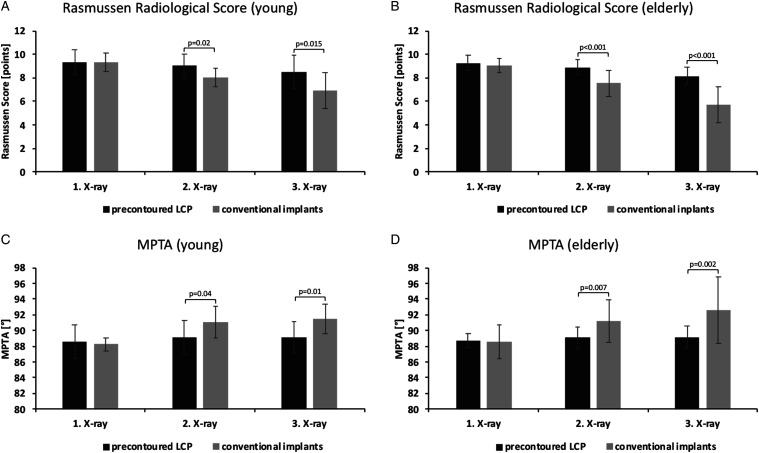
The radiological outcomes by age groups after implant-specific subgrouping revealed significantly impaired results for conventional implants in young patients (a, c). The effect was even more pronounced in elderly with highly significant deterioration for conventional implants (b, d).

The mean RRS value was 9.3 ± 1.0, 9.0 ± 1.0, and 8.5 ± 1.5 on the first, second, and third X-ray in the precontoured LCP subgroup compared to 9.3 ± .8, 8.0 ± .8, and 6.9 ± 1.6 in the conventional implant subgroup. On the second and third X-ray, the precontoured LCP subgroup featured significantly higher RRS values compared to the conventional implant subgroup (*P* =.02 and .015, respectively; Figure 4A). The according MPTAs were 88.6 ± 2.2, 89.1 ± 2.2, and 89.1 ± 2.1° in the precontoured LCP and 88.2 ± .8, 91.1 ± 2.0, and 91.5 ± 1.9° in the conventional implant group. The MPTA was significantly higher in the conventional implant group and the second and third X-ray compared to the precontoured LCP group (*P* = .04 and .01, respectively; Figure 4C). Based on the third X-ray, the distribution of the nominal RRS results (excellent, good, fair, and poor outcome) did not differ significantly between the 2 subgroups (precontoured LCP: 8, 4, 1, and 0 vs conventional implants: 2, 7, 3, and 1; *P* = .09).

### Precontoured LCP Versus Conventional Implants in Elderly Patients

Implant-specific subgrouping of the elderly patients revealed ORIFs utilizing precontoured LCP in 20 and conventional implants in 13 cases. Again here, there were no significant differences between the 2 subgroups regarding age, gender distribution, the mean size and depression depth of the lateral joint surface fragments, calculated void volume or frequency of void filling (Table 4). The mean RRS value was 9.3 ± .6, 8.9 ± .7, and 8.2 ± .8 on the first, second, and third X-ray in the precontoured LCP subgroup compared to 9.0 ± .6, 7.5 ± 1.1, and 5.7 ± 1.6 in the conventional implant subgroup. On the second and third X-ray, the precontoured LCP subgroup featured significantly higher RRS values compared to the conventional implant subgroup [*P*<.001 in both cases; Figure 4(b)]. The according MPTAs were 88.7 ± .9, 89.1 ± 1.4, and 89.2 ± 1.4° in the precontoured LCP and 88.6 ± .9, 91.2 ± 2.7, and 92.6 ± 4.2° in the conventional implant group. The MPTA was significantly higher in the conventional implant group and the second and third X-ray compared to the precontoured LCP group [*P* = .007 and .002, respectively; Figure 4(d)]. Based on the third X-ray, the distribution of the nominal RRS results (excellent, good, fair, and poor outcome) differed significantly between the 2 subgroups (precontoured LCP: 5, 15, 0, and 0 vs conventional implants: 0, 4, 6, and 3; *P* < .001).

## Discussion

This is the first study revealing superior radiological outcome measures in AO41B3-fractures after ORIF utilizing precontoured LCP compared to conventional implants, in both young and elderly patients. The effects are significant in young and even more pronounced in elderly patients. Strongpoints of the study are exclusively investigating split-depression fractures to the lateral tibial plateau, comparable CT-morphological fracture characteristics (area and maximum depression depth of the joint surface fragments, calculated bony void volume) and void filling rates across the implant-specific subgroups as well as a sufficient statistical power for the separated evaluation in young and elderly patient cohorts.

To date, the literature has shown rather conflicting data on the potential benefit in outcome measures when utilizing LCP in split-depression fractures. The data situation is thereby confounded by studies insufficiently separating the heterogeneous patient subgroups underlying the bimodular age distribution pattern of split-depression fractures as well as by studies investigating mixed tibial plateau fracture entities, even though split-depression fractures often represent the majority of cases. The bimodular age distribution pattern is due to mainly male young patients and predominately female elderly patients. For the present study, this was shown in a larger population of 104 patients with AO41B3-fractures and ORIF prior to applying the exclusion criteria (Figure 2) as well as in the cohorts of finally included patients (Table 3). Comparable observation in populations of mixed tibial plateau fractures entities (Schatzker I-VI) were reported by others.^2,12,20^ Despite the fact that the prevalence of osteoporosis is significantly higher in elderly patients^2^ and that the proximal tibia represents a predilection site of osteoporosis-associated loss of bone mineral density, trabecular connectivity, and subchondral cortical bone thickness,^3,4^ no significant differences regarding the area of the depressed joint surface fragments, the maximum depression depth, and the calculated bony void volume were detected when comparing the groups of young and elderly patients (Table 3). Furthermore, no significant differences regarding the radiological outcome measures at final follow-up were evident when comparing young and elderly patients prior to implant-specific subgrouping (Figure 3). It is noteworthy in the context that the frequency of void filling during the ORIF was significantly higher in the elderly group (*P* = .02). The frequency of void filling in the treatment of tibial plateau fractures varies considerably cross the available studies.^21–25^ Due to a lack of well-designed studies, the specific effects of void filling on the outcome after tibial plateau fractures has not yet been precisely determined. Furthermore, a clear consensus and mandatory recommendations regarding the indication for void filling are still missing. Against this background, it is difficult to evaluate the effect of the void filling frequency in the presented groups and subgroups with regards to the radiological outcome.

Tahririan et al. investigated pooled tibial fractures with a high ratio of split-depression fractures comparing nonlocking and locking plate fixations in a young population (mean age 34.5 years in both groups, predominately male patients).^25^ The authors reported on a higher rate of postoperatively increasing MPTA resulting in valgus malalignment and associated with inferior functional outcome in the nonlocking group. In contrast, the group of Abghari et al investigated a mid-aged population (mean age 48.4 years, 51.9% male) and found that the clinical and radiographic outcomes of split-depression fractures treated with nonlocking and locking plates were similar.^21^ A recently published pooled age-matched pair analyses (mean age 50.1 and 51.3, predominately male patients) on split-depression fractures revealed that precontoured LCP prevent the subsidence of the reduced joint surface fragments more sufficiently and allow for improved patient outcomes compared to conventional implants.^17^ A previous study demonstrated that inferior radiological outcomes approximately 1 year after the ORIF of split-depression fractures to the lateral tibial plateau are associated with inferior clinical mid-term outcomes.^17^ The group of Shimizu et al reported on radiological outcome measures of 23 elderly patients with heterogeneous tibial plateau fractures and ORIF.^24^ Utilizing locking plates in 21 and conducting a void filling in 16 of the case resulted in 6 excellent, 11 good, 4 fair, and no poor nominal RSS. The according mid-term clinical outcomes in these patients were comparably satisfying.

The actual statistically significant differences in radiological outcome measures of the present study were only revealed after implant-specific subgrouping of both age groups. After a mean interval of 65.4 ± 38.0 weeks, young patients showed a mean RRS value of 8.5 ± 1.5 for precontoured LCP compared to 6.9 ± 1.6 for conventional implants (*P* =.015). Around this time (66.0 ± 33.5 weeks), elderly patients featured a highly significant difference in the mean RRS value with 8.2 ± .8 for precontoured LCP and 5.7 ± 1.6 for conventional implants (*P* < .001). There was a high ratio of ORIF with simultaneous void filling, but the ratio was comparable across the implant-specific subgroups in young as well as in elderly patients (Table 4). In both age groups, the utilization of conventional implants was associated with a pronounced subsidence of the lateral joint line and a higher valgus angulation as revealed by the MPTA. In young patients with conventional implants, the MPTA increased from 88.2 ± .8° immediately postoperatively to 91.5 ± 1.9° at final follow-up. At the same time, the MPTA in elderly patients increased from 88.6 ± .9° to 92.6 ± 4.2°. Evaluating the distribution of nominal RRS results did not show statistically significant differences when comparing the implant-specific subgroups in young patients but revealed highly significant superior radiological outcomes in elderly patients with precontoured LCP compared to conventional implants (*P* < .001).

Limitations of the present study are the restricted level of evidence and general shortcomings due to the retrospective cohort study design. Furthermore, it is a general limitation of radiological outcome studies that reliable conclusions on the functional outcome cannot be drawn. Future randomized studies with prospective data collection and patient-reported outcome measures are required to validate the present findings. Although, the ongoing change in common practice with an increasingly widespread application of precontoured LCP in ORIF of tibial plateau fractures may already jeopardize the popular support for such studies.

## Conclusion

The utilization of precontoured LCP in the treatment of AO41B3-fractures is associated with improved radiological outcome measures. This effect is significant in young but even more pronounced in elderly patients. Consequently, precontoured LCP should closely be considered for ORIF of any AO41B3-fracture, but especially in elderly patients.
